# Developing Biodegradable Films from Mango (*Mangifera indica*) Starch and Extract: A Rheological and Physical Study

**DOI:** 10.3390/gels11100825

**Published:** 2025-10-14

**Authors:** Santander E. Lastra-Ripoll, Luis Mieles-Gómez, David Ramirez-Brewer, Ronald Marsiglia-Fuentes, Somaris E. Quintana, Luis A. García-Zapateiro

**Affiliations:** Research Group of Complex Fluid Engineering and Food Rheology, Universidad de Cartagena, Cartagena 130015, Colombia; slastrar@unicartagena.edu.co (S.E.L.-R.); lmielesg@unicartagena.edu.co (L.M.-G.); dramirezb1@unicartagena.edu.co (D.R.-B.); rmarsigliaf@unicartagena.edu.co (R.M.-F.); squintanam@unicartagena.edu.co (S.E.Q.)

**Keywords:** starch films, kernel starch, peel extracts, mechanical properties, rheological properties

## Abstract

The development of biodegradable films with antioxidant properties offers a promising approach to food preservation. This study focused on creating and characterising mango starch-based films enriched with mango peel extract (MPE) at concentrations of 0, 1, and 2%, using peels from mangoes (*Mangifera indica* var. Corazon) at organoleptic maturity, obtained as residual byproducts (peel and seed) for active food packaging applications. An MPE extraction yield of 35.57 ± 2.74% was achieved using ultrasound-assisted extraction (UAE), confirming its rich phenolic content and antioxidant activity as a natural alternative to synthetic preservatives. Rheological analysis revealed that the films exhibited pseudoplastic behavior, with complex viscosity reducing as angular frequency increased. Incorporating MPE at concentrations up to 1% enhanced the films’ viscoelastic properties, while a 2% addition significantly altered their frequency and temperature dependence. The rheological modeling showed that the fractional Maxwell model with two springpots described the films more accurately than the generalized Maxwell model. This approach offered a clearer understanding of their viscoelastic response, especially under changes in frequency and temperature. Mechanical characterization indicated that adding MPE improved film strength while reducing solubility. Although film thickness remained unchanged, increasing MPE concentration led to greater opacity and darker coloration. These changes offer advantages in food packaging by enhancing UV protection and reducing oxidative degradation. Crucially, the incorporation of MPE significantly increased the phenolic content and antioxidant capacity of the films, as confirmed by ABTS assays. These findings strongly support the potential of MPE-based films for active packaging, providing a sustainable and functional alternative for preserving light-sensitive food products. Among the tested formulations, films with 1% MPE demonstrated the most effective balance of rheological stability, mechanical strength, and antioxidant capacity.

## 1. Introduction

In recent years, the search for sustainable solutions in food packaging has gained significant importance due to environmental issues associated with non-biodegradable plastics and the increasing demand for fresh products with extended shelf life [1]. In this context, active packaging has attracted attention for its functional properties, which not only act as a physical barrier, but also actively contribute to food preservation [2,3]. Furthermore, recent advances in food packaging technology emphasise the importance of “smart” packaging that can monitor and respond to changes in the internal atmosphere of the packaging [4]. Such systems can release antioxidants or antimicrobial agents when needed, providing an additional layer of protection as the food ages [5]. This concept has been explored in the development of packaging materials incorporating pH-sensitive or moisture-responsive components, allowing for the packaging to adjust its properties according to the internal environment [6]. In addition, the increasing consumer demand for clean-label products and sustainable solutions has reinforced interest in natural-based active packaging systems [7]. As a result, the integration of natural extracts into biodegradable films has become a major area of research, aligned with both environmental sustainability and consumer health preferences [8].

Among the biopolymers explored for active packaging, agro-industrial residues of mango (*Mangifera indica*) have demonstrated potential as functional ingredients [9]. Their incorporation into packaging materials provides multiple benefits, including biodegradability, reduction in plastic pollution, cost-effectiveness through residue valorisation, and the addition of bioactive compounds with antioxidant and antimicrobial activity. Mango starch, obtained from fruit processing byproducts, has proven to be particularly suitable as a base material for packaging applications [10]. When combined with natural extracts rich in phenolic compounds, such as those obtained from mango peel, including gallic acid, catechins, quercetin, and mangiferina films, can gain additional functional activity [11]. Similar advances have been reported with other biopolymer formulations, such as Aloe vera-infused starch–protein composites that extend the shelf life of fresh fruit [12], or bilayer starch systems functionalized with alliin to achieve pH-responsive antimicrobial effects [13].

Mango peel extract (MPE) has been increasingly investigated as a natural preservative. Edible coatings enriched with MPE have been shown to delay lipid oxidation and inhibit microbial growth in meat and fish products [14], while its incorporation into films has also extended the shelf life of fresh fruits [15]. These findings highlight the potential of MPE as a multifunctional additive for active packaging. Similarly, mango starch has been studied as a biodegradable polymer matrix for active packaging, demonstrating improved moisture barrier properties and the preservation of sensitive food components. Blends of mango starch with bioactive extracts have been reported to enhance mechanical resistance while maintaining antioxidant activity. The use of biopolymers, therefore, not only reduces dependence on petroleum-derived plastics, but also aligns with the principles of circular economy by valorising agricultural waste streams [16].

Despite these promising results, scaling these technologies from laboratories to industrial applications remains a major challenge. Variability in plant-derived extract composition, controlling the migration of bioactive molecules into food, and maintaining desirable sensory attributes are issues that are still discussed in the scientific community. An important aspect under continuous discussion is the potential migration of phenolic compounds from active packaging into food matrices, particularly as biodegradable films degrade over time. Although controlled migration can provide antioxidant and antimicrobial benefits, excessive release may raise regulatory and safety considerations that need to be addressed in future applications [17]. Mango starch, a biodegradable polysaccharide derived from agricultural byproducts, emerges as an excellent base material for developing active packaging [18]. This material is abundant, cost-effective and capable of forming films with favourable mechanical and barrier properties [19]. However, pure starch has limitations, such as its sensitivity to moisture and its limited thermal stability. To address these challenges, researchers have explored the incorporation of natural extracts rich in phenolic compounds with antioxidant activity to improve functionality and expand applications [20]. Several studies have reported various biological properties of mango extract, including antimicrobial, antioxidant, and other bioactive effects [21]. Globally, research is increasingly focusing on plant-derived extracts as alternatives to synthetic chemicals used in food preservation, which are associated with long-term health risks [22].

The objective of this study was to rheologically characterise and model film-forming solutions based on mango starch and natural mango peel extract and subsequently develop and comprehensively evaluate the physical properties of the resulting films. This research aims to offer a sustainable and efficient packaging alternative for the food industry, in line with global trends toward functional and environmentally friendly materials.

## 2. Results and Discussion

### 2.1. Mango Peel Extract—MPE

The extraction yield of the mango peel extract (MPE) was 35.57 ± 2.74%. This yield was higher than that reported by Dorta et al. [23] for mango peel extracts (var. Keitt) using microwave-assisted extraction with an ethanol-water solvent (6–14%), and by Mieles-Gómez et al. [10] for extracts from mango peels (var. Fachir) using ultrasound-assisted extraction (7.02%). Furthermore, this result falls within the range reported by Ramirez-Brewer et al. [24] for mango peel extracts (var. Sugar) using microwave-assisted extraction with an ethanol-water solvent (31.98–58.81%). However, it was lower than the yields reported by Guandalini et al. [25] for mango peel extracts (var. Tommy Atkins) using ultrasound-assisted extraction with an ethanol-water solvent (41–73%), and by Vélez-Erazo et al. [26] for extracts from mango peel varieties Criollo, Hilaza, and Corazón (41.62–62.80%). Extraction yield primarily depends on the nature and composition of the plant peel, the solvent used, and the extraction method [27]. In this study, the high yield can be attributed to the enhanced mass transfer effect caused by ultrasonic waves. These waves induce powerful bubble implosions in the solvent, a phenomenon that converts potential energy into heat and lowers the solvent’s viscosity. As a result, the solvent can penetrate the plant matrix more effectively, improving the overall extraction process [28].

The total phenolic content (TPC) of the MPE was 121.33 ± 11.20 mg GAE g^−1^ of mango peel (MP) dry matter (DM). This value was significantly higher than those reported for other Colombian varieties, such as Tommy and Sugar (80.94 to 124.70 mg GAE g^−1^ extract DM) [29], as well as for Brazilian Tommy Atkins mangoes (6 to 13.82 mg GAE g^−1^ MP DM) [25] and Indian Alphonso mangoes (49.89 to 69.84 mg GAE g^−1^ MP DM) [30]. However, the TPC was comparable to the value reported for Spanish varieties (Keitt, Kent, and Osteen) at 103.82 mg GAE g^−1^ DM [31], and to a previous extract from mango var. Corazón (101.52 ± 7.38 mg GAE g^−1^ DM) obtained with a 50% ethanol-water solvent [32]. The antioxidant activity of the MPE, measured by ABTS radical scavenging, was 508.67 ± 25.72 µmol TE g^−1^ MP DM. This result is consistent with findings for by-products of the Haden mango variety (239.13 to 1155.83 µmol Trolox g^−1^ MP DM) [33] and is substantially higher than the activity observed in Tommy Atkins mango extracts (46.7 to 73.8 µmol Trolox g^−1^ MP DM) [34]. This strong antioxidant activity can be attributed to the high TPC, which is closely linked to its ability to combat oxidative stress [35,36]. Moreover, the synthesis of secondary metabolites and their biological properties are known to vary depending on factors like plant species, cultivar, soil conditions, post-harvest handling, and maturity stage [37]. The extraction conditions can further influence the biological activity of the extracts.

### 2.2. Mango Starch

The extraction of mango kernel starch (MKS) yielded 44.05 ± 2.58%, with an apparent amylose content of 28.46 ± 0.93%. These values are similar to those reported by Ferraz et al. [38] and Mieles-Gómez et al. [39], with yields ranging from 39.35% to 44.95% for mango varieties Ubá and Corazón, respectively. In contrast, significantly higher yields have been reported for other varieties, including Safeda, Chausa, and Dussheri (59.17–61.58%) [40], as well as for Amrapali and Surjapuri (52.89–62.11%) [41]. This variability in yield can be attributed to differences in the mango’s origin, variety, agronomic conditions, and the extraction methods employed. When compared to other non-conventional sources, the yield of mango kernel starch is notably high, surpassing that of avocado seed starch (19.54%) [42] and litchi seed starch (40.7%) [43].

The apparent amylose content of the MKS is consistent with the 27.28% reported for mango seed starch var. Fachir [10] and higher than the values for var. Sugar (18.61%) and var. Tommy (22.71%) [44]. Compared to conventional starches, the amylose content of MKS is higher than that reported for cassava (18.9%) [45], potato (21%) [46], corn (25%) [47], and wheat starch (2.41–27.93%) [48]. This is significant because amylose plays a key role in retrogradation and the formation of resistant starch—desirable properties for developing food products with a lower glycemic index. Starches with higher amylose content exhibit a greater tendency for gelation and retrogradation, leading to more resistant gels with improved textural properties, making them valuable for applications such as thickeners, stabilizers, and coatings [49].

### 2.3. Film Forming Solution (FFS)

#### Rheological Properties of FFS and Modelling

**Figure 1:**
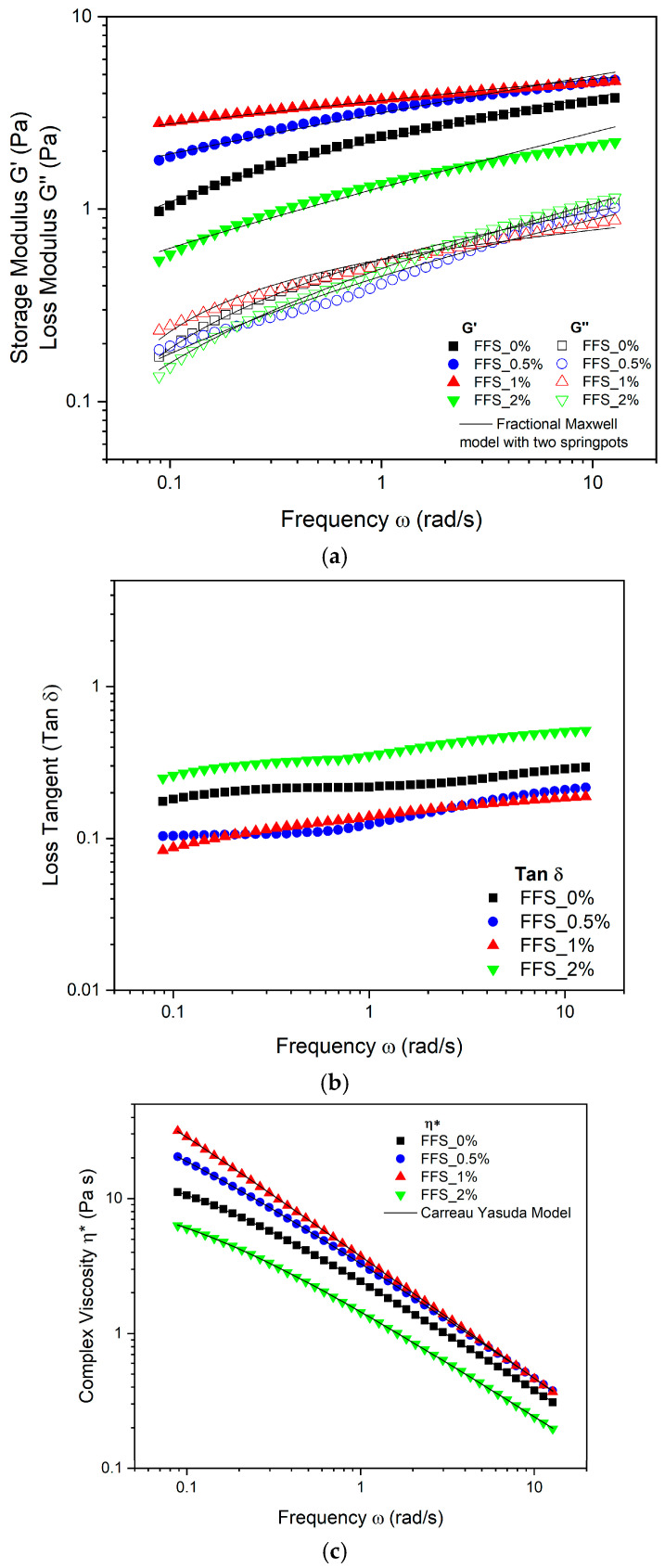
Viscoelastic properties for film forming solutions (FFS) with mango peel extract (0; 0.5; 1; 2%). (**a**) Storage modulus $\left(G^{\prime }\right)$ and modulus $\left(G^{\prime \prime }\right)$. (**b**) Loss tangent (Tan δ). (**c**) Complex viscosity $\eta^{*}$ as a function of frequency (ω).

Figure 1 shows the viscoelastic properties of the film-forming solutions (FFS) with added MPE. As presented in Figure 1a, the storage modulus $\left(G^{\prime }\right)$ and the loss modulus ($G^{\prime \prime })$ were measured as a function of angular frequency $\left(\omega \right).$ Throughout the entire mechanical spectrum, the elastic component $\left(G^{\prime }\right)$ of the FFS was greater than the viscous component $\left(G^{\prime \prime }\right)$ for all samples. This indicates that the FFSs exhibit a solid-like behavior typical of these systems, as the entanglement of polymeric chains traps MPE as a functional component in a dispersed phase, forming strong intermolecular bonds [50]. An increase in MPE concentration from 0% to 1% led to a rise in both $G^{\prime }$ and $G^{\prime \prime }$ moduli. However, at a 2% concentration, both values decreased, suggesting that FFS with 2% MPE will behave more like liquids during shorter mixing processes. Consequently, the energy required to deform the material may be lower in FFS containing more than 2% MPE [51]. This behavior can be attributed to polysaccharide–polyphenol interactions, which initially form a stronger network and enhance viscoelastic properties. Beyond a critical concentration; however, these properties may decrease due to a weakening of the starch network from excessive interactions [24].

Figure 1b displays the tan (δ) values for the FFS. With all values below 1 (Tan δ<1), the results indicate that elastic behavior predominates over viscous behavior $\left(G^{\prime }>\;G^{\prime \prime }\right)$. Specifically, the tan (δ) values for FFS_0.5% and FFS_1% were closer to zero, indicating a more pronounced elastic nature, as a purely elastic material has a δ = 0° [52]. The complex viscosity curves, shown as a function of angular frequency in Figure 1c, were fitted to the Carreau-Yasuda model, which provided an optimal fit ($R^{2}>0.99$ and $F_{min}\approx 0$). The model’s fitted parameters are presented in Table 1. The results show that all FFS exhibit pseudoplastic (shear-thinning) non-Newtonian fluid behavior (*n* < 1), evidenced by a decrease in complex viscosity with increasing angular frequency. Comparable results were reported by [24,53] for edible coatings with xanthan gum and hydrogels containing mango extracts, respectively. The zero-shear viscosity ($\eta_{0}^{*}$) increased with MPE concentration up to 1%, but decreased at the 2% level. This may be due to a higher polyphenol concentration weakening the MKS network structure, thereby reducing viscosity [54]. Similarly, the relaxation time (λ) increased up to a 1% MPE concentration before decreasing at 2%. The initial increase is likely due to stronger polysaccharide-polyphenol interactions limiting chain mobility [55], while the subsequent decrease may be caused by a weakening of these interactions, potentially due to pH changes from the phenolic acids in the MPE [24]. The parameter ‘a’ of the Carreau-Yasuda model appeared to be independent of the MPE concentration. Similar results have been reported by Lastra Ripoll et al. [53] and Bertolo et al. [56] for film-forming solutions made with xanthan gum containing natural mango extracts and starch/gelatin with acerola and açaí extracts, respectively.

Furthermore, the experimental data from the mechanical spectrum were fitted to generalized Maxwell models (N=1, 2, 3) and fractional Maxwell models with one and two springpots. As shown in Table 2, the mean square error function ($F_{min}$) reveals that the fractional Maxwell model with two springpots provided the best fit, presenting the lowest $F_{min}$ values (close to 0). This result is consistent with findings by Ramirez-Brewer et al. [57], who demonstrated that fractional rheological models can describe the viscoelastic behavior of food samples more accurately than classical models, often with an equal or fewer number of parameters.

Table 3 presents the fitted parameters of the fractional Maxwell model. The addition of MPE significantly (p<0.05) affected the elastic modulus ($G^{\prime }$), which increased with MPE concentrations up to 1% (FFS_1%), but decreased at 2% (FFS_2%). This suggests the formation of bonds between MPE polyphenols and MKS polymer chains via hydroxyl (−OH) or carboxyl (−COOH) groups. However, at excessive concentrations, these interactions may disrupt the gel network and weaken the system [27], making the viscous component more prominent, as observed for FFS_2% in Figure 1a where the gap between the $G^{\prime }$ and $G^{\prime \prime }$ moduli narrows. These rheological findings correlate with the physical properties of the films. The FFS with a higher *G*′ modulus and η_0_* (FFS_1%) exhibited greater tensile strength, whereas the FFS with lower values (FFS_2%) showed greater elongation. This can be explained by the principle that greater elasticity and rigidity in the FFS result in higher tensile strength, while lower elasticity and rigidity lead to more extensible films [58]. The relaxation time (τ), related to the memory effects of the FFS, increased from 243.676 s to 355.830 s with the addition of 0% to 2% MPE, likely due to increased interactions between MPE polyphenols and the MKS polymer network [24,27]. Ultimately, the mechanical analysis revealed that incorporating 1% MPE increased tensile strength and reduced solubility, suggesting strong interactions between phenolic compounds and starch chains that reinforce the polymer network. Similar trends have been observed in cassava and corn starch films enriched with natural extracts [15]. Conversely, the 2% MPE formulation showed reduced strength and higher solubility, indicating that excessive phenolic loading can disrupt network cohesion, a phenomenon also reported in other bioactive-enriched starch films [59].

**Figure 2:**
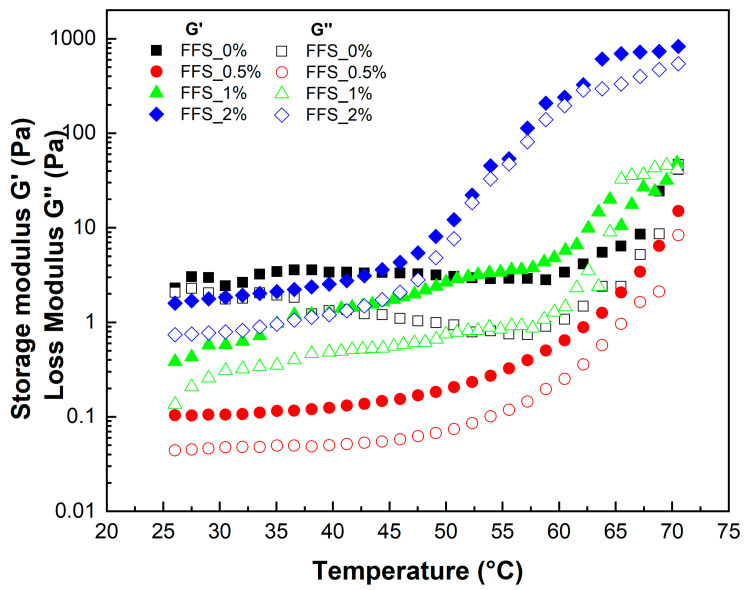
Storage modulus $\left(G^{\prime }\right)$ and modulus $\left(G^{\prime \prime }\right)$ in function of temperature.

The temperature-dependent viscoelastic properties are shown in Figure 2. Across the entire temperature range studied (25 °C to 70 °C), all FFS formulations with MPE behaved as gels, as evidenced by $G^{\prime }$ values remaining greater than $G^{\prime \prime }\;$ values. Both moduli increased with temperature, suggesting a reinforcement of the polymeric structure as the gel matrix transitions from a weak to a stiff state during heating. This is likely due to the strengthening of leached amylose by starch granules [60]. Parameters from the temperature sweep are listed in Table 4. The sol–gel transition temperature (Ts) ranged from 58.94 °C to 62.58 °C for the 0%, 0.5%, and 1% MPE formulations. However, for FFS_2%, the Ts value was significantly lower at 42.79 °C, indicating that a 2% MPE concentration lowers the gelation point. This may be attributed to MKS chains organizing around the MPE to form a cubic structure, which alters the reinforcement temperature of the gel matrix [61]. In such a structure, dispersed water molecules provide volume and plasticity. Upon heating, water loss from the system causes an increase in both $G^{\prime }$ and $G^{\prime \prime }$ moduli [62]. This effect enhances the elasticity of the FFS, as higher temperatures reduce hydrogen bonding and accelerate polymer chain movement by removing solvating water molecules [63].

### 2.4. Films

#### 2.4.1. Film-Forming Capacity of Solutions

The films with added MPE were easily removed from the casting panels, displaying a uniform surface appearance and a continuous matrix. It was observed that the films containing MPE were smoother than the control film (without MPE), which exhibited a rougher surface. In all cases, no bubbles were visible on the film surfaces.

#### 2.4.2. Physical Properties of Films

Table 5 presents the physical properties of the films. Film thickness showed no significant differences (p> 0.05) among samples with increasing MPE concentration. This consistency can be attributed to all film solutions being adjusted to the same volume [64]. Similar results were reported by Choi et al. [65], who observed that adding strawberry extracts did not affect the thickness of chitosan/gelatin/poly(vinyl alcohol) films. The tensile strength (TS) of the films ranged from 1177.24 to 1816.71 Pa, with a significant increase (p<0.05) observed as more MPE was incorporated. This increase is linked to the strengthening of the biopolymer chains by phenolic compounds [66]. The reinforcement of films by polyphenol addition has been previously documented; for instance, films formulated with various polyphenolic extracts showed a significant increase in TS with higher polyphenol content, confirming that phenolic compounds consolidate the biopolymer network [67]. Regarding elongation at break (EAB), significant differences (p<0.05) were observed between the control film and the MPE-containing films. However, there were no significant differences (p>0.05) in EAB among the films with varying MPE concentrations. This can be explained by interactions between starch and phenolic acids, which may result in a reinforced matrix with a mild elasticizing effect [68]. Similar results were reported by Xue et al. [67], who found that adding polyphenols increased the EAB of Chitosan/Zein films. In contrast, Choi et al. [65] reported that the addition of strawberry extracts to Chitosan films decreased the EAB. The water solubility of the films decreased significantly (p<0.05) for the active films compared to the control. These findings indicate that MPE concentration substantially affects film water solubility, which decreased by more than half. This markedly lower solubility suggests that crosslinking agents in the MPE lead to a denser structural arrangement within the starch polymer matrix. Films with reduced solubility are ideal for preserving food products and extending their shelf life [69]. The enhanced mechanical properties of these films can also be attributed to polyphenol-polymer cross-linkage through sulfide and noncovalent bonding, which creates a well-structured three-dimensional network [70].

#### 2.4.3. Optical Properties of Films

The optical properties of films containing MPE are summarized in Table 6. The luminosity $\left(L^{*}\right)$ of the samples decreased significantly (p < 0.05) with the addition of MPE, indicating a progressive darkening as the extract concentration increased. This can be attributed to the phenolic compounds in the extract, which possess conjugated structures capable of absorbing specific wavelengths of light, thereby reducing reflectance and decreasing transparency [71]. Furthermore, the formation of bonds between these bioactive compounds and the polymer matrix can induce changes in light scattering, intensifying the darkening effect [72]. The color parameters $a^{*}$ (+, red; −, green) and $b^{*}$ (+, yellow; −, blue) serve as indicators of how the films might influence the final color of their application matrix [73]. The $a^{*}$ value (red-green hue) decreased in the FFS_0.5% sample, imparting a subtle greenish tint not easily perceptible to the naked eye. In contrast, the ${\;b}^{*}$ value (yellow-blue hue) increased with higher extract concentrations, a finding consistent with previous studies on the impact of natural extracts on polymer matrices [74]. The total color difference ($∆E^{*}$) increased significantly with MPE concentration, which is related to the differential scattering of light caused by aggregates of phenolic compounds, leading to variations in opacity and spectral absorption [75]. Previous research has shown that a higher content of polyphenols and carotenoids in films can modify the visual perception of color, causing the material to exhibit a darker, more intense tone [76]. Similarly, opacity values showed a significant increase (p<0.05) with MPE addition. This property is beneficial for active packaging, as the modified color and reduced light transmission can improve the stability of light-sensitive foods and serve as a visual indicator of antioxidant activity [77].

#### 2.4.4. Total Phenolic Compounds (TPC) and Antioxidant Activity (AA)

The antioxidant capacity of the films was evaluated by quantifying Total Phenolic Compounds (TPC) and assessing ABTS free-radical scavenging activity. As shown in Table 7, TPC values ranged from 0.19 to 4.65 mg GAE g^−1^ of film. The results indicate that incorporating MPE was effective, as increasing its concentration led to significantly higher TPC values (p<0.05), directly reflecting the TPC content of the extract. Similar findings have been reported for gelatin-based films [78], chitosan/starch films [79], and antioxidant chitosan films with various extracts [80], as well as for films incorporating other natural extracts like green algae [81] and green tea [82]. The ABTS assay results (Table 6) revealed that the control film (FFS_0%) exhibited an antioxidant activity of 0.28 ± 0.01 μmol Trolox g^−1^ of film. With the addition of MPE, this activity increased substantially—by more than 70-fold—to values of 10.52 ± 0.10, 16.86 ± 0.45, and 20.16 ± 0.27 μmol Trolox g^−1^ of film for 0.5%, 1%, and 2% MPE, respectively. This enhancement correlates with the concentration of phenolic compounds, which can interrupt oxidation chain reactions, donate hydrogen atoms, and scavenge free radicals [74]. This trend has also been observed in edible films containing green algae extract [81], multilayer films with green tea extract [82], and active films with bee bread extract [83]. Consistent with the high phenolic content of mango peel extract [84], the film with the highest MPE concentration (FFS_2%) exhibited the greatest radical-scavenging capacity. However, while higher concentrations provided greater antioxidant activity, they also compromised the films’ mechanical and rheological performance. Therefore, the 1% MPE formulation offered the best balance, combining sufficient antioxidant potential with improved structural stability. Similar trade-offs between antioxidant activity and physical properties have been noted in other starch-based films enriched with polyphenolic extracts [59].

## 3. Conclusions

This study successfully demonstrated that ultrasound-assisted extraction is an effective method for obtaining mango peel extract (MPE) with a high yield (35.57 ± 2.74%), significant phenolic content, and robust antioxidant activity, confirming its potential as a natural alternative to synthetic preservatives. The extracted mango kernel starch, characterized by a high apparent amylose content, proved to be a valuable ingredient for creating stable food formulations requiring enhanced gelling properties. The incorporation of MPE into starch-based films (FFS) resulted in pseudoplastic, solid-like viscoelastic behavior ($G^{\prime }\;>\;G\prime \prime$,). Key viscoelastic properties were enhanced at MPE concentrations up to 1%; however, a decrease was observed at a 2% concentration, while temperature sensitivity increased. The experimental data were accurately described by the fractional Maxwell and Carreau-Yasuda models, which optimally fitted the frequency-dependent $G^{\prime }$, $G^{\prime \prime }$, and $\eta^{*}$, data, respectively. Functionally, the MPE-enriched films exhibited improved mechanical properties, such as increased tensile strength and reduced water solubility. The films also showed a progressive darkening with higher extract concentrations, a beneficial trait for UV protection in food packaging. Most importantly, the incorporation of MPE significantly enhanced the films’ phenolic content and antioxidant activity. Overall, these findings strongly suggest that MPE-based films are a promising biodegradable solution for developing active packaging to preserve light-sensitive food products.

## 4. Materials and Methods

### 4.1. Materials

Mango fruits (*Mangifera indica* var. Corazon) were cultivated in the Department of Bolívar (Colombia) and were harvested at commercial maturity (stage 4). Ethanol (99.5% purity), sodium bisulfite and sodium carbonate anhydrous (99.5% purity) were purchased from Panreac (Barcelona, Spain). The gallic acid standard (>98% purity), Folin–Ciocalteu reagent, and (±)-6-hydroxy-2.5.7.8-tetramethyl-chromane-2-carboxylic acid (Trolox, 97% purity), were purchased from Sigma–Aldrich (St. Louis, MO, USA).

Mango fruits (*Mangifera indica*) were washed with an aqueous solution of sodium hypochlorite (100 ppm), then the peel, pulp and seed were manually removed. Subsequently, the peel was dried using a freeze-dryer (Labconco Freezone 1.5 L, Kansas City, MO, USA) and the seeds were dried at 40 °C for 4 h in a hot air tray dryer to facilitate the separation of the kernel and the hull; then the kernel was separated from the hull, cut and dried in a hot air tray dryer at 40 °C for 12 h. Finally, the size was reduced in a mill (IKA MF 10.1 Germany cutting-grinding head) to obtain a powder with particle size less than 250 µm.

### 4.2. Ultrasound Assisted Extraction of Mango Peel Extract

Ultrasound-Assisted Extraction (UAE) was carried out following the methodology describe by Mieles-Gómez et al. [10] with some modifications. An Ultrasonic Processor (SX Sonic, FS-1200N, Shanghai, China) operating at a frequency of 60 kHz and an input power of 240 W was used. The extraction process involved mixing mango peel powder with a hydroethanolic solution (75:25 ethanol: water) at a 1:10 ratio, maintaining a temperature of 40 °C for 20 min. The resulting mixture was then filtered and dried using a rotary evaporator, followed by freeze-drying. Extraction yields (Ys %) were determined using Equation (1):

(1) $$Ys\%=\frac{mango\;peel\;extract\;(g)}{mango\;peel\;(g)}\times 100$$

### 4.3. Determination of Total Phenolic Compounds and Antioxidant Capacity

The total phenolic content (TPC) of the mango peel extract expressed as mg of gallic acid equivalents (GAE) g^−1^ dry matter (DM) of mango peel (MP) was determined following the method of describe by Singleton et al. [85]. Briefly, 50 uL of extract will be added to 3 mL of MilliQ water and 250 uL of Folin–Ciocalteu reagent, and stirred for 5 min. Luego, this mixture is added with 750 uL of calcium carbonate solution at 20% *w*/*w* and 950 uL of MilliQ water and left to rest in the dark for 2 h at 25 °C. Absorbance at 760 nm was subsequently measured.

The antioxidant capacity (AA) of the mango peel extract, specifically its ability to scavenge ABTS free radicals, was assessed using the method outlined by Re et al. [86]. The ABTS^⁎+^ radical cation was generated by mixing the ABTS stock solution (7 mM) with 2.45 mM potassium persulfate after incubating the mixture at 25 °C for 16 h in the dark. Once the ABTS^⁎+^ radical was formed, the absorbance of the solution was adjusted to 0.700 ± 0.02 at 734 nm using ethanol in a Genesys 10S UV-vis spectrophotometer (Thermo Fischer Scientific Inc., Waltham, MA, USA). Subsequently, 990 uL of ABTS solution was added to 10 uL of the extract sample, and the mixture was allowed to stand at 25 °C in the dark until the absorbance reached a plateau. Absorbance was measured at 734 nm. Trolox was used as the reference standard, and the results were expressed as µMol Trolox equivalent (TE) g^−1^ DM of MP.

### 4.4. Isolation of Mango Kernel Starch

The isolation of mango kernel starch (MKS) was carried out following the methodology described by Mieles-Gómez et al. [39] with some modifications. A suspension of mango kernel flour (1:10 *w*/*v* ratio) was prepared in a 1% sodium bisulfite solution and stirred magnetically at room temperature for 4 h. The mixture was then homogenized using an Ultra-Turrax (IKA T25, Staufen, Germany) and filtered through a 100-mesh sieve. The residue was thoroughly washed with distilled water, while the filtration was left to settle for 30 min, after which the supernatant was decanted. The starch residue was resuspended in distilled water and centrifuged at 3000× *g* for 10 min to precipitate the starch. Finally, the mango kernel starch was dried in a hot air tray dryer at 40 °C for 12 h.

### 4.5. Determination of Apparent Amylose

The apparent amylose content was determined following the Morrison et al. [87] colorimetric method; the readings were taken at 620 nm on a Genesys 10S UV-vis spectrophotometer (Thermo Fischer Scientific Inc., Waltham, MA, USA), which was also used to produce a straight-line standard curve obtained from pure amylose solutions.

### 4.6. Preparation of Film-Forming Solutions

Film-forming solutions (FFS) were prepared following the method described by [88] with some modifications. A 5% mango kernel starch dispersion was immediately prepared by dissolving the starch in 100 mL of distilled water and heating it in a water bath at 90 °C in magnetic stir and heating plate (IKA RCT Basic, Staufen, Germany) for 30 min under constant stirring at 1100 rpm. The solution was then cooled to 60 °C, and glycerol (25% of the starch weight) was added while maintaining continuous stirring for 15 min. After that, the mixture cooled to 40 °C, and mango peel extracts (MPE) were incorporated at 0.5% (MPE_0.5%), 1.0% (MPE_1.0%), and 2% (MPE_2%); also, cooling of a sample without MPE was performed (MPE_0%). The FFS were then homogenized using an Ultra-Turrax homogenizer (T-25 basic IKA, Staufen, Germany) at 7500 rpm for 3 min. After preparation, the film-forming solutions were rheologically characterized.

### 4.7. Rheological Characterization and Modelling of the FFS

The rheological characterization of FFS was performed using a modular advanced rheometer system Mars 60, Haake (Thermo Scientific, Karlsruhe, Germany), equipped with a serrated plate-plate geometry (35 mm diameter and 0.5 mm diameter GAP) to prevent slip effects. Samples were allowed to equilibrate for 300 s prior to the rheological characterization to ensure uniform thermal and mechanical history.

#### 4.7.1. Stress Sweep

To determine the linear viscoelastic regime (LVR) for all samples, stress amplitude sweep tests were conducted over a range of 0.001 to 1000 Pa at an angular frequency of 0.1 Hz and a temperature of 25 °C.

#### 4.7.2. Frequency Sweep

The frequency sweep was performed at 0.01 Pa to maintain the stress in the LVR, within the range 10^−1^–10^1^ rad s^−1^ [9].

#### 4.7.3. Temperature Sweep

The thermo-viscoelastic properties were evaluated using a temperature ramp from 25 to 70 °C at a constant frequency of 0.1 Hz within the LVR, with a heating rate of 5 °C min^−1^.

Dynamic data were obtained from oscillatory shear experiments, including the storage modulus ($G^{\prime }$), which represents the elastic component, and the loss modulus ($G^{\prime \prime }$), which corresponds to the viscous component of the material. Additionally, complex viscosity $\eta^{*}$ and the loss tangent (Tan δ), defined as the ratio $G^{\prime \prime }/\;G^{\prime }$, were measured to assess the balance between elastic and viscous responses as a function of frequency and temperature [53].

#### 4.7.4. Rheological Modelling

For analyzing the viscoelastic behavior of all the samples, it was modeled using the following models [55,57]:

Carreau-Yasuda model. This model allows us to describe the behavior of complex viscosity $\eta^{*}\;$ as a function of angular velocity ω.

(2) $$\eta^{*}\left(\omega \right)=\eta_{o}^{*}{\left(1+{\left(\lambda \omega \right)}^{a}\right)}^{\frac{n-1}{a}}$$

where $\eta_{o}^{*}$ is zero complex viscosity in Pa s, λ is the relaxation time in s, a represents the width of the transition region between Newtonian and power-law behavior and n is the flow index (0<n<1: pseudoplastic fluid behavior).

**Figure 3:**
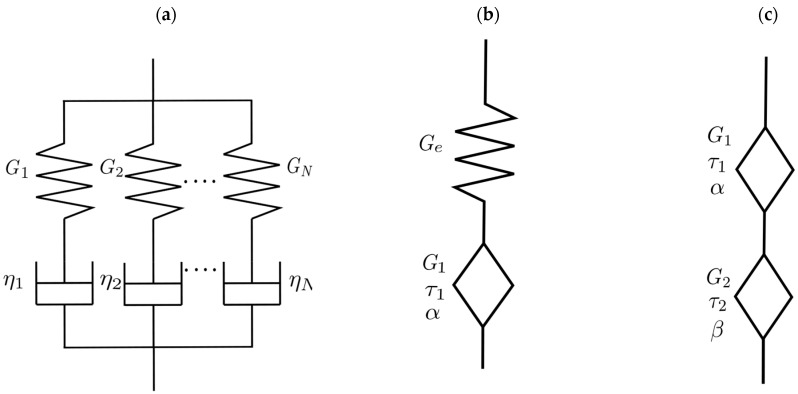
Rheological model (**a**) Generalized Maxwell model. (**b**) Fractional Maxwell model with one springpots. (**c**) Fractional Maxwell model with two springpots.

Generalized Maxwell model: This model consists of multiple Maxwell elements (a spring and a damper connected in series) connected in parallel as shown in Figure 3a. The equations of the modules $G^{\prime }$ and $G^{\prime \prime }$ as a function of the angular frequency ω of the generalized Maxwell model are shown below:

(3) $$G^{\prime }(\omega )=\;\sum_{i=1}^{N}G_{i}\frac{({\tau_{i}\omega )}^{2}\;}{1\;+\;({\tau_{i}\omega )}^{2}}$$

(4) $$G^{\prime \prime }(\omega )=\sum_{i=1}^{N}G_{i}\frac{\tau_{i}\omega \;}{1+({\tau_{i}\omega )}^{2}}$$

where $G_{i}$ is the elastic modulus in Pa of the i element, τ  is the characteristic time of the model in s given by $\frac{\eta_{i}}{G_{i}}$, with the viscosity in Pa·s of the  i element, and N is the number of elements in the model.

Fractional Maxwell model: These models consist of a spring element and a Scott-Blairs element or springpots, and two Scott-Blairs elements or springpots connected in series as shown in Figure 3b,c. The fractional Maxwell model with a springpot presents the following equations (Equations (4) and (5)) for the modules $G^{\prime }\;$ and $G^{\prime \prime }$ as a function of the angular frequency ω:

(5) $$\begin{array}{r} G^{\prime }(\omega )=G_{e}\frac{\left(\omega \tau {)}^{2\alpha }+(\omega \tau {)}^{\alpha }cos(\pi \alpha /2\right)}{1+2(\omega \tau {)}^{\alpha }cos(\pi \alpha /2)+(\omega \tau {)}^{2\alpha }} \end{array}$$

(6) $$\begin{array}{r} G^{\prime \prime }(\omega )=G_{e}\frac{\left(\omega \tau {)}^{\alpha }sin(\pi \alpha /2\right)}{1+2(\omega \tau {)}^{\alpha }cos(\pi \alpha /2)+(\omega \tau {)}^{2\alpha }}\; \end{array}$$

where $G_{e}$ is the elastic modulus in Pa, τ  is the characteristic time of the model in s given by following expression $\tau^{\alpha }=\frac{G_{1}\tau_{1}}{G_{e}}$. For the fractional Maxwell model with two springpots, the equations for the modules $G^{\prime }$ and $G^{\prime \prime }$ as a function of the angular frequency ω are shown below:

(7) $$\begin{array}{r} G^{\prime }(\omega )=G\frac{\left(\omega \tau {)}^{\alpha }cos(\pi \alpha /2)+(\omega \tau {)}^{2\alpha -\beta }cos(\pi \beta /2\right)}{1+2(\omega \tau {)}^{\alpha -\beta }cos(\pi (\alpha -\beta )/2)+(\omega \tau {)}^{2(\alpha -\beta )}} \end{array}$$

(8) $$\begin{array}{r} G^{\prime \prime }(\omega )=G\frac{\left(\omega \tau {)}^{\alpha }sin(\pi \alpha /2)+(\omega \tau {)}^{2\alpha -\beta }sin(\pi \beta /2\right)}{1+2(\omega \tau {)}^{\alpha -\beta }cos(\pi (\alpha -\beta )/2)+(\omega \tau {)}^{2(\alpha -\beta )}}\; \end{array}$$

where G is the elastic modulus in Pa and τ  is the characteristic time of the model in s given by following expression $G=\;G_{1}{\left(\frac{\tau_{1}}{\tau }\right)}^{\alpha }$, and $\tau =\;{\left(\frac{G_{1}\tau_{1}^{\alpha }}{G_{2}\tau_{2}^{\beta }}\right)}^{\frac{1}{\alpha -\;\beta }}$, with α>β.

The Vortex Search Algorithm (VSA) was used to estimate the parameters of the rheological models, by minimizing the mean square error function $F_{min}$:

(9) $$F_{min}=\;\sum_{k=1}^{M}{\left(\frac{\eta^{*}\left(\omega_{k}\right)-\;\eta_{k}^{*}}{\eta_{k}^{*}}\right)}^{2}\;\;$$

(10) $$\begin{array}{cc} F_{min}= & \sum_{k=1}^{M}\left({\left(\frac{G^{\prime }\left(\omega_{k}\right)-G_{k}^{\prime }}{G_{k}^{\prime }}\right)}^{2}+{\left(\frac{G^{\prime \prime }\left(\omega_{k}\right)-G_{k}^{\prime \prime }\;}{G_{k}^{\prime \prime }}\right)}^{2}\right)\;\;\\ subject\;to & 0<\beta <\alpha <1 \end{array}$$

where $G^{\prime }\left(\omega_{k}\right)$, $G^{\prime \prime }\left(\omega_{k}\right)$ and $\eta^{*}\left(\omega_{k}\right)$ are the values predicted by rheological models (Equations (2)–(8)), $G_{k}^{\prime }$, $G_{k}^{\prime \prime }$ and $\eta_{k}^{*}$ are the experimental values, and k number data. The MATLAB 2024a software was used to implement the Vortex Search Algorithm (VSA) [57].

### 4.8. Preparation of Film

The films were prepared using the casting method, following the methodology proposed by Niu et al. [89]. For this, 15 mL of the film-forming solution (FFS) containing 0.5% (FFS_0.5%), 1.0% (FFS_1.0%), 2% (FFS_2%) of MPE, and without MPE (FFS_0%) were poured into polycarbonate trays (6 cm × 6 cm) to obtain films of uniform thickness, which were dried in an oven at 40 °C for 48 to 72 h and stored in a desiccator until characterization.

### 4.9. Film Characterization

#### 4.9.1. Thickness

Thickness of films was measured using a Mitutoyo IP65 digital micrometer (Mitotuyo, Tokyo, Japan). Measurements were taken at six random positions for each treatment, and the average values were used to calculate the film’s tensile strength.

#### 4.9.2. Mechanical Properties

The tensile strength (TS) and elongation at break (EAB) of each film were measured using an EZ-SX Food Texture Analyzer equipped with a 50 kg load cell (Shimadzu, Kyoto, Japan) according to the standard method ASTM D882-91 (American Society for Testing and Materials) [90]. Five film strips (20 mm × 60 mm) were prepared and placed onto grip pairs with an initial separation of 30 mm, which were then attached to the equipment, the samples were stretched at a speed of 50 mm min^−1^. The TS and EAB were calculated using Equation (11) and Equation (12), respectively:

(11) $$TS=\frac{F_{max}\;(N)}{A\;(m^{2})}$$

where $F_{max}$ is the maximum force applied before failure (N) and A is the cross-sectional area of the sample.

(12) $$EAB\left(\%\right)=\frac{l_{max}\;}{l_{0}\;}\times 100$$

where $l_{max}$ the film elongation (mm) is the moment of rupture, and $l_{0}$ is the initial grips length (mm) of sample.

#### 4.9.3. Optical Properties

The color values of the films were determined with a colorimeter (Konika Minolta CR-20, Konica Minolta, Inc., Tokyo, Japan). The values of the parameters of lightness $\left(L^{*}\right)$, red-green chromaticity $\left(a^{*}\right)$, and blue-yellow chromaticity $\left(b^{*}\right)$ were obtained from readings with the colorimeter. Equation (13) was used to calculate color variation $\left({∆E}^{*}\right)$.

(13) $$∆E^{*}={\left[{\left({∆L}^{*}\right)}^{2}+{\left({∆a}^{*}\right)}^{2}+{\left({∆b}^{*}\right)}^{2}\right]}^{0.5}$$

The opacity of the films was determined using a Genesys 10S UV–vis spectrophotometer (Thermo Fischer Scientific Inc., Waltham, MA, USA). The films were cut to 20 mm pieces and directly placed in a cell for the measurements. An empty test cell was used as the reference. The opacity was calculated using Equation (14).

(14) $$Opacity=\frac{{Abs}_{600}}{d}$$

where ${Abs}_{600}$ is the absorbance at 600 nm and d is the thickness of each film.

#### 4.9.4. Solubility

The water solubility (WS) of each film was evaluated according to the method described by [91] with some modifications. Briefly, the films were cut into 20 mm × 20 mm pieces, dried at 45 ° C for 30 min. Subsequently, the weight of each dry film ($W_{0}$) was taken and soaked in 80 mL of distilled water in an Erlenmeyer flask. The flasks were placed on a magnetic stir plate (IKA RCT Basic, Germany) for 1 h at 600 rpm. The films were then dried at 100 °C in an oven until reaching constant weight ($W_{1}$). The water solubility was estimated using Equation (15):

(15) $$WS\;(\%)=\frac{W_{0}-W_{1}}{W_{0}}\;\times \;100$$

where *WS* is the water solubility expressed as percent, and $W_{0}$ and $W_{1}$ are initial weight of films and final weight of dried films, respectively.

#### 4.9.5. Determination of Total Phenolic Compounds and Antioxidant Activity

The extraction of measurable phenolic compounds from the films was performed by [92]. The total phenolic content (TPC) and the ability to scavenge ABTS free radicals of films were determined according to the method described in Section 4.3.

### 4.10. Statistical Analysis

The data were analyzed with unidirectional ANOVA and Fisher’s LSD test using Statgraphics software (version centurion XVI) to determine statistically significant differences (*p* < 0.05) between the samples. The coefficient of variation (C.V) was used to analyze the relationship between the standard deviation and the means. All tests were performed in triplicate and measurements were expressed as mean ± standard deviation.

## Figures and Tables

**Table 1:** Estimated parameters of Carreau–Yasuda model.

Sample Code	$\eta_{0}^{*}$	λ	a	n	$R^{2}$	$F_{min}$
FFS_0%	20.831 ^a^	11.968 ^a^	0.968	0.164	0.999	$2.22\times 10^{-4}$
FFS_0.5%	156.388 ^b^	64.697 ^b^	0.578	0.106	0.999	$8.69\times 10^{-5}$
FFS_1%	199.723 ^c^	79.858 ^c^	1.164	0.092	0.999	$7.91\times 10^{-4}$
FFS_2%	12.529 ^d^	13.093 ^d^	0.912	0.192	0.999	$1.18\times 10^{-3}$

$\eta_{0}^{*}$: Zero complex viscosity; λ: Relaxation time; a: Transition parameter between Newtonian and power-law behavior; n: Flow index. Data with C.V < 0.05. Different letters in the same column express statistically significant differences (p <0.05).

**Table 2:** Minimum values F_min for the Generalized Maxwell and Fractional Maxwell model.

Sample Code	Rheological Model				
	Maxwell *N* = 1	Maxwell *N* = 2	Maxwell *N* = 3	Maxwell One Springpots	Maxwell Two Springpots
FFS_0%	48.704	4.891	4.854	3.860	0.109
FFS_0.5%	48.888	3.263	3.640	6.247	0.277
FFS_1%	47.761	3.192	3.073	2.735	0.160
FFS_2%	51.440	4.553	1.095	2.605	0.249

**Table 3:** Estimated parameters of the fractional Maxwell model with two springpots.

Sample Code	G	τ	α	β
	(Pa)	(s)		
FFS_0%	1.182 ^a^	225.795 ^a^	0.635	0.164
FFS_0.5%	1.744 ^b^	243.676 ^b^	0.385	0.154
FFS_1%	2.051 ^c^	286.830 ^c^	0.753	0.107
FFS_2%	0.308 ^d^	355.968 ^d^	0.739	0.269

G:  Elastic modulus; τ: Characteristic time; α, β: Fractional exponents. Data with C.V < 0.05. Different letters in the same column express statistically significant differences (p <0.05).

**Table 4:** Rheological properties of Film-forming solutions (FFS) with mango peel extract (0; 0.5; 1; 2%) during temperature sweeps.

Sample Code	$T_{s}$	$G_{p}^{\prime }$	$G_{p}^{\prime \prime }$
	(°C)	(Pa)	(Pa)
FFS_0%	60.48 ^a^	46.85 ^a^	41.42 ^a^
FFS_0.5%	58.94 ^b^	15.02 ^d^	8.35 ^b^
FFS_1%	62.58 ^c^	48.32 ^c^	45.83 ^c^
FFS_2%	42.79 ^d^	826.52 ^d^	542.51 ^d^

*Ts:* Temperature at which $G^{\prime }$ increased drastically. $G_{p}^{\prime }$: The maximum value of $G^{\prime }\;$ during heating. $G_{p}^{\prime \prime }$*:* The maximum value of $G^{\prime \prime }$ during heating. Data with C.V < 0.05. Different letters in the same column express statistically significant differences (p <0.05).

**Table 5:** Physical properties of films with bioactive compounds from mango peel extract.

Sample Code	Thickness (mm)	TS (Pa)	EAB (%)	WS (%)
FFS_0%	0.277 ± 0.018 ^a^	1177.24 ± 58.17 ^a^	27.58 ± 6.46 ^a^	29.77 ± 1.07 ^a^
FFS_0.5%	0.247 ± 0.046 ^a^	1395.88 ± 29.28 ^b^	46.61 ± 1.42 ^b^	18.14 ± 1.19 ^b^
FFS_1%	0.239 ± 0.028 ^a^	1740.36 ± 37.92 ^c^	46.77 ± 0.81 ^b^	15.57 ± 1.50 ^c^
FFS_2%	0.239 ± 0.045 ^a^	1816.71 ± 60.65 ^c^	50.33 ± 4.88 ^b^	13.53 ± 1.19 ^c^

TS: Tensile strength; EAB: Elongation at break; WS: Water solubility. Data are the mean ± standard deviation. Different letters in the same columns express statistically significant differences (p <0.05).

**Table 6:** Optical properties of films with bioactive compounds from mango peel extract.

Sample Code	$L^{*}$	$a^{*}$	$b^{*}$	$∆E^{*}$	Opacity
FFS_0%	56.98 ± 1.74 ^a^	13.10 ± 0.83 ^ac^	26.60 ± 1.86 ^a^	-	2.45 ± 0.20 ^a^
FFS_0.5%	52.55 ± 2.30 ^b^	8.02 ± 0.38 ^b^	34.90 ± 0.68 ^b^	11.05 ± 0.77 ^a^	2.90 ± 0.24 ^b^
FFS_1%	41.85 ± 1.63 ^c^	14.23 ± 0.42 ^a^	38.20 ± 0.95 ^c^	19.16 ± 0.39 ^b^	3.30 ± 0.05 ^c^
FFS_2%	35.45 ± 2.48 ^d^	12.53 ± 1.30 ^c^	38.39 ± 1.41 ^c^	24.64 ± 3.09 ^c^	3.95 ± 0.05 ^d^

Data are the mean ± standard deviation. Different letters in the same columns express statistically significant differences $\left(p\;<0.05\right).$

**Table 7:** Total phenolic compounds (TPC) and antioxidant activity (AA) of films with bioactive compounds from mango peel extract.

Sample Code	TPC	AA
	(mg GAE g^−1^ of Film)	(µMol Trolox g^−1^ of Film)
FFS_0%	0.19 ± 0.03 ^a^	0.28 ± 0.01 ^a^
FFS_0.5%	1.90 ± 0.19 ^b^	10.52 ± 0.10 ^b^
FFS_1%	3.60 ± 0.10 ^c^	16.86 ± 0.45 ^c^
FFS_2%	4.65 ± 0.09 ^d^	20.16 ± 0.27 ^d^

Data are the mean ± standard deviation. Different letters in the same columns express statistically significant differences (p <0.05).

## Data Availability

The original contributions presented in this study are included in the article. Further inquiries can be directed to the corresponding author.

## Abbreviations

MPE	Mango peel extract
UAE	Ultrasound-assisted extraction
TPC	Total phenolic content
ABTS	Ability to scavenge
WS	Water solubility
TS	Tensile strength
EAB	Elongation at break
